# Characterization and application of a novel *Campylobacter* phage CC_R7 as a biocontrol agent in chicken meat

**DOI:** 10.1016/j.crfs.2025.101182

**Published:** 2025-08-20

**Authors:** Muhammad Shahzad Rafiq, Jie Chen, Muhammad Akmal, Dongxin Ma, Ali Asif, Junhao Wang, Shuaifeng Gu, Yufeng Gu, Pan Tao, Haihong Hao

**Affiliations:** a National Key Laboratory of Agricultural Microbiology, Huazhong Agricultural University, Wuhan, China; b National Reference Laboratory of Veterinary Drug Residues, Huazhong Agricultural University, Wuhan, 430070, China; c Huazhong Agricultural University, Shenzhen Institute of Nutrition and Health, Shenzhen, 518000, China; d Shihezi University, Shihezi City, Xinjiang, 832000, China; e Department of Fisheries and Aquaculture, University of Veterinary and Animal Sciences, Lahore, 54000, Pakistan

**Keywords:** Bacteriophage, *Campylobacter*, Stability, Biofilm removal, Chicken meat

## Abstract

***Campylobacter*** is a major foodborne pathogen, commonly transmitted through poultry. The emergence of multidrug-resistant strains due to antibiotic overuse in poultry farming highlights the need for monitoring resistance patterns and exploring alternative control strategies, such as bacteriophage application. This study examined the antimicrobial resistance patterns in *Campylobacter jejuni* (*C. jejuni*) and *Campylobacter coli* (*C. coli*). Additionally, a novel lytic *Campylobacter* phage CC_R7 was isolated, characterized, and subjected to complete genomic analysis. The phage CC_R7 application was evaluated for biofilm removal under slaughterhouse conditions and as a biocontrol agent in chicken meat. The results showed that the resistance rate in *C. coli* was higher than in *C. jejuni.* The phage CC_R7 has a genome size of 180,566 bp, and no virulent gene was found. It has a broad host range, killing 60 % of *C. coli* and 27.2 % of *C. jejuni* tested strains with excellent adsorption, a short latent period of 40 min, and a high burst size of 119 virions. The phage remained stable across temperatures (4 °C–50 °C) and pH levels (4–10). Moreover, phage CC_R7 has the potential to inhibit biofilm formation and reduce *Campylobacter* contamination in chicken meat by 1.2 log/g. Therefore, *Campylobacter* phage CC_R7 has unique characteristics to combat multidrug-resistant *Campylobacter* strains and can be used as a feed additive for biocontrol in food.

## Highlights

• In China, antimicrobial resistance in *C. coli* and *C. jejuni* is increasing.
• The *Campylobacter* phage CC_R7 is capable of lysing both *C. coli* and *C. jejuni.*
• The Phage CC_R7 has significant genetic and biological potential to combat *Campylobacter.*
• It effectively reduces biofilm formation and *Campylobacter* load in chicken meat.

## Graphical abstract

**Figure:**
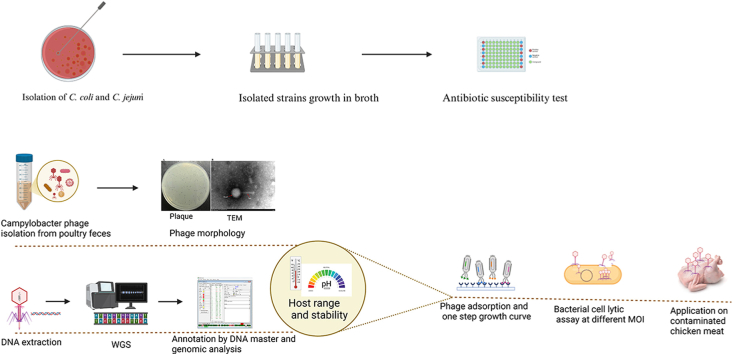

## 1 Introduction

*Campylobacter* is a foodborne pathogen that causes campylobacteriosis. Domestic animals like sheep, goats, cattle, chickens, and pets are the main reservoirs of this bacterium (Sahin et al., 2017). The World Health Organization (WHO) identifies *Campylobacter* as a leading cause of diarrhea because of its resistance to multiple antibiotics (Olson et al., 2022). The *Campylobacter* genus consists of more than 20 species; however, *Campylobacter jejuni* (*C. jejuni*) and *Campylobacter coli* (*C. coli*) are the most common *Campylobacter* species that cause human gastrointestinal illnesses (Alam et al., 2006). *Campylobacte*r is mainly transferred to humans by ingesting contaminated food, while poultry meat is the primary transmission source (50–80 %) (El-Saadony et al., 2023). Globally, *Campylobacter* infects 96 million people annually (Damtew et al., 2024), with the highest incidence reported in the Czech Republic (215 cases per 100,000 in 2019), followed by Australia (146.8 per 100,000 in 2016) and New Zealand (126.1 per 100,000 in 2019) (Liu et al., 2022). The Centers for Disease Control and Prevention (CDC) estimates that *Campylobacter* infection affects 1.5 million people annually in the United States (Al Hakeem et al., 2025). According to the European Union's One Health 2023 Zoonosis Report, campylobacteriosis was the leading cause of human zoonotic diseases, accounting for 148,181 cases, which represented over 58 % of all reported zoonotic diseases (Authority, E.F.S., Prevention, E.C.f.D., Control, 2024). In China, *Campylobacter* has been increasingly reported. A study conducted in Wenzhou between 2017 and 2019 showed a 10.5 % detection rate in diarrheal patients, the highest in 15 years (Zhang et al., 2020). A 2010 risk assessment estimated 118 cases per 100,000 people from poultry consumption. Recent outbreaks, including five foodborne incidents in schools in Wenzhou between 2021 and 2022, further highlight the growing threat of *Campylobacter* in China (Li et al., 2025). Recently, antibiotic resistance in *Campylobacte*r has been increasing, threatening public health (Tang et al., 2021). It is reported that clinical isolates of *Campylobacter* from China exhibit strong resistance to various antibiotics, including quinolones and tetracycline (Gao et al., 2023).

Different methods, like adding organic acid to drinking water, cleaning the carcass, improving biosecurity, cleaning after slaughter, and reducing environmental exposure, have been researched to lower *Campylobacter* in the food supply. However, some of these methods are not very effective or are costly to implement (Umaraw et al., 2017). Recently, bacterial antimicrobial resistance (AMR) has focused on employing bacteriophages to manage multidrug-resistant (MDR) bacteria (Askoura et al., 2021). Bacteriophages are viruses that prey on bacteria, and the environmental richness of phages is exceptionally high, with an estimated total number of phages of approximately 10^31^ particles (Mushegian, 2020). Phages have many advantages over antimicrobials; they can kill specific pathogenic bacteria without disturbing the normal microflora and destroying bacterial biofilms, which play a vital role in bacterial virulence and persistence (Bolocan et al., 2019). In therapeutic applications, phages have been used to target and eradicate pathogens like *Acinetobacter baumannii*, *Staphylococcus aureus*, and *Pseudomonas aeruginosa* (Schooley et al., 2017; Jault et al., 2019). In the food production sector, the Food and Drug Administration (FDA) has approved certain phage-based products as Generally Recognized As Safe (GRAS). The majority of approved phages targeting foodborne pathogens are aimed at *Listeria monocytogenes*, *Salmonella*, and Shiga toxin-producing *Escherichia coli* (STEC) (FDA, 2019, 2022).

*Campylobacter* phages belong to the *Myoviridae* or *Siphoviridae* families (Javed et al., 2014). They have been divided into three groups according to the size of their genomes: group I, which is estimated to be 320 kb; group II, which is 180–190 kb; and group III, which is 130–140 kb (Sails et al., 1998). Group I *Campylobacter* phages haven't been studied much or used yet, but some group II phages, such as *Campylobacter* phage CP220, *Campylobacter* phage CPt10, and *Campylobacter* phage CP21, have been fully sequenced, while *Campylobacter* phage vB_CcoM-IBB_35 (IBB_35) is listed in GenBank with five different pieces (Timms et al., 2010; Carvalho et al., 2012; Hammerl et al., 2012). A notable characteristic of group II phages is their high propensity to infect *C. jejuni* and *C. coli* (Sails et al., 1998). The genome of Group II *Campylobacter* phages possesses genes encoding several components, including *clamp loader*, *membrane proteins*, *T4-like tail proteins*, *transposases*, and metabolic enzymes. Notably, they also harbor up to twelve proteins containing S-adenosylmethionine (SAM) domains, which are not found in group III phages (Hammerl et al., 2012). Furthermore, group II *Campylobacter* phages exhibited a certain degree of similarity to T4-type (*Escherichia* phage) phages at the protein level (Timms et al., 2010).

Studies have demonstrated that *Campylobacter* bacteriophages can effectively reduce *Campylobacter* on food items like broilers, chicken skin, and meat. However, limited data are available on the biological and genomic characterization of these phages, and so far, only one *Campylobacter* phage product (GRN 966) has been approved by the FDA (FDA, 2020; Ushanov et al., 2020). Therefore, to combat multidrug-resistant (MDR) *Campylobacter* in the food chain, a *Campylobacter* bacteriophage with a broad host range and well-characterized biological and genomic properties is needed to decrease the load of both *C. coli* and *C. jejuni* in food products and animals.

This study aimed to assess the antibiotic resistance profiles of *C. coli* and *C. jejuni* strains and to isolate a novel *Campylobacter* phage capable of combating MDR *Campylobacter* strains. The phage was thoroughly characterized, including its complete genomic sequence and lytic efficacy. Additionally, the application of phage for biofilm removal under slaughterhouse conditions and in chicken meat was tested to control *Campylobacter* contamination.

## 2 Materials and methods

### 2.1 Bacterial strains isolation and antibiotic susceptibility test

A total of 120 strains of *C. coli* and 33 strains of *C. jejuni* were isolated from fecal samples collected at poultry farms in Hubei, Hunan, and Jiangxi Provinces of China from 2019 to 2022. Modified Skirrow agar (Hopebio Technology Co., Ltd.) was used to isolate *Campylobacte*r (Butcher and Stintzi, 2017). Suspected *Campylobacter* colonies were grown in brucella broth (Becton, Dickinson and Company, Sparks, MD 21152 USA) and subjected to multiplex Polymerase Chain Reaction (PCR) to confirm *C. coli* and *C. jejuni* (Denis et al., 1999). The *Campylobacter* isolates were grown on Columbia blood agar (Oxoid Ltd, Wade Road Basingstoke, Hants, RG24 8 PW, UK) supplemented with 5 % sheep blood (Hongquan Bio) at 42 °C under microaerobic conditions (5 % O_2_, 10 % CO_2_, and 85 % N_2_) for 24–48 h, then *Campylobacter* colony was grown in brucella broth and stored at −80 °C for further use. Nine antibiotics, including gentamycin (32 μg/mL), clindamycin (16 μg/mL), erythromycin (64 μg/mL), florfenicol (64 μg/mL), tetracycline (64 μg/mL), ciprofloxacin (64 μg/mL), azithromycin (64 μg/mL), nalidixic acid (64 μg/mL), and telithromycin (8 μg/mL), were tested to see how sensitive the isolated *C. coli* and *C. jejuni* strains. The antibiotic concentrations were chosen based on Clinical and Laboratory Standards Institute (CLSI) breakpoints for *Campylobacter* species. Antibiotic susceptibility testing was performed by using the broth microdilution method according to CLSI (Azrad et al., 2018).

### 2.2 Bacteriophage isolation, purification, and propagation

A total of 48 fecal samples from poultry farms in Hubei province were collected and transported to the National Reference Laboratory of Veterinary Drug Residues, Huazhong Agricultural University. The *Campylobacte*r phage was isolated as described previously with slight modifications (Gencay et al., 2016). Briefly, 3 g of a poultry feces sample was mixed in 10 mL SM buffer (Phygene, 50 mM Tris HCl, 8 mM MgSO_4_, 100 mM NaCl, gelatin 0.01 %, pH 7.5) and kept overnight in a shaker at 4 °C. Next, the samples were centrifuged at 10,000 rpm for 10 min, and the supernatant was filtered through a 0.22 μm filter membrane (Merck Millipore Ltd). The obtained filtrate was mixed with an equal amount of 2 × brucella broth for enrichment, and 100 μL of each *Campylobacter* isolate was added to the filtered sample (Supplementary File 1, Table S1). The sample was incubated at 42 °C overnight and then centrifuged at 10,000 rpm for 10 min; the supernatant was filtered through a 0.22 μm filter membrane. In the next step, mixed 400 μL freshly cultured indicator strain *C. coli* (99-2) with 4 mL NZCYM broth (Sangon Biotech) containing 0.5 % agar (Biofroxx). Then, poured the mixture gently on the top of the NZCYM-agar (1.2 % agar) plate, and dried it. Subsequently, spot 10 μL of the enriched sample and incubated at 42 °C under microaerobic conditions for 24–48 h. The formation of plaques was observed. After confirming the inhibition zone, the single plaque was picked up using a 200 μL pipette tip and resuspended in a 500 μL SM buffer. Each single plaque was purified 3 times on an NZCYM double agar plate by plaque assay (Townsend et al., 2021). Phage was propagated by the complete lysate plate method, according to Bonilla et al. (2016). Phage titer was determined by plaque assay and stored at 4 °C for further experiments.

### 2.3 Phage purification by cesium chloride density gradient

For purification of phage, 100 mL phage lysate was centrifuged at 200,000×*g* for 2 h in Optima XPN-100 (Beckman Coulter). The supernatant was discarded, and the pellet was resuspended in 2 mL SM buffer. Next, the phage suspension was purified by cesium chloride (CsCl) density gradient (Tao et al., 2016). Phage band was collected and dialyzed in dialysis buffer I (10 mM Tris HCL 1.211g, 20 mM NaCl 11.7g, 50 mM Mgcl_2_ 1.0165, water 1 L, pH 7.5) for 5 h and in dialysis buffer II (10 mM Tris HCL 1.211g, 20 mM NaCl 2.925g, 50 mM Mgcl_2_ 1.0165, water 1 L, pH = 7.5) overnight. The purified phage was collected in a glass vial, and after measuring the titer, the phage was stored at −80 °C for further use.

### 2.4 Electron microscopic examination

A 10 μL cesium chloride-purified phage sample (∼10^9^ PFU/mL) was absorbed on a copper grid and negatively stained with phosphotungstic acid (2 %, pH 7.0) for 15 s (Wang et al., 2020). After that, the phage morphology was examined by using transmission electron microscopy (HITACHI H-7650, Tokyo, Japan) at 80 kV.

### 2.5 Phage DNA extraction, sequencing, *de novo* assembly, and annotation

Phage DNA was extracted by using a commercially available phage DNA extraction kit (Norgen Biotek Corp. Phage DNA isolation kit), according to the manufacturer's instructions. The China National Gene Bank (CNGB) conducted genomic DNA library preparation and whole genome sequencing. Briefly, a 300 bp DNA fragment library was prepared using the MGIEasy DNA Library prep kit (V1.0) and sequenced using a sequencer (DNBSEQ-T7; MGITech, China). Subsequently, the raw reads obtained post-sequencing were inspected with FastQC v 0.12.1 (Andrews, 2010), revealing 3,456,734 read pairs with an average length of 150 bp. The reads were further processed with Fastp v 0.23.2 (Chen et al., 2018) with default settings for quality control, adapter trimming, and quality filtering. To simplify the *de novo* assembly process and avoid unnecessary complexities, the filtered raw reads were randomly sub-sampled using seqtk (https://github.com/lh3/seqtk) to ensure coverage of 100X (Rihtman et al., 2016). The sub-sampled reads were then *de novo* assembled using Unicycler v 0.5.0 (Wick et al., 2017), resulting in 2 contigs (151,711 bp and 28,016 bp). The gap between the 2 contigs was filled by PCR (Supplementary File 1, Table S2), resulting in a single contig. The obtained single contig was circularized by a closure PCR (https://cpt.tamu.edu/training-material/topics/de-novo-assembly/tutorials/genome-close-reopen/tutorial.html) (Supplementary File 1, Table S3), resulting in 180,566 bp, and re-opened starting from the terminase protein (Ye et al., 2006).

Further, DNA Master v5.23.6 (a freely available tool) was used for sequence analysis and annotation (Pope and Jacobs-Sera, 2018). Where Glimmer and GeneMark were used for Open reading frame (ORF) prediction (Besemer and Borodovsky, 2005; Delcher et al., 2007). Aragon and tRNA scan were used to predict tRNAs (Lowe and Eddy, 1997; Laslett and Canback, 2004). The putative functional assignment for each ORF was conducted by analyzing the corresponding amino acid sequence from the National Center for Biotechnology (NCBI), conserved domain database (CDD), BLASTp, and HHpred using the default parameters (McGinnis and Madden, 2004; Söding et al., 2005; Marchler-Bauer et al., 2010). The whole genomic sequence of *Campylobacter* phage CC_R7 was submitted to GenBank, and the accession number PQ092953 was assigned.

### 2.6 Phage taxonomic classification and genome comparison

The major capsid protein and terminase protein of *Campylobacter* phage CC_R7, along with other similar protein sequences searched in BLASTp hits, were used for phylogenetic analysis. The ClustalW (Thompson et al., 2003) was used to build multiple sequence alignments (MSAs), which served as input for creating a maximum likelihood phylogenetic tree in IQ-TREE v2.3.5 (http://iqtree.cibiv.univie.ac.at/). The tree was constructed with 1000 rapid bootstrap replications (Hoang et al., 2018). The genomic nucleotide sequence similarity of phage CC_R7 to other sequenced phages was determined using a heatmap generated with the VIRDIC tool (https://viridic.icbm.de). The comparative genomic analysis of *Campylobacter* phage CC_R7 with other *Campylobacter* phages (CP220, CP20, CP21), the BLASTn top hits, was conducted using BLASTn in Easyfig v.2.2.3 (Sullivan et al., 2011).

### 2.7 Phage adsorption and one-step growth curve

The phage adsorption assay was performed as previously described with slight modifications (Rattanachaikunsopon and Phumkhachorn, 2006). Briefly, phage lysate (2.62 × 10^7^ PFU/mL) was infected with the host or indicator strain *C. coli* (99-2) at Multiplicity of Infection (MOI) 0.1 and incubated at 42 °C while shaking. The samples were taken at 5-min intervals up to 25 min, centrifuged at 10,000 rpm for 10 min, and filtered through a 0.22 μm filter membrane. Unabsorbed bacteriophage was determined by measuring the titer of the filtrate using the double agar plate method. For a one-step growth curve, 2 mL bacterial host solution containing 10^8^ CFU/mL was mixed with a 2 mL bacteriophage suspension containing 10^8^ PFU/mL. The mixture was then incubated at 42 °C for 15 min. Subsequently, the combination underwent centrifugation at 10,000 rpm for 15 min. The resulting pellet was then dissolved in 10 mL of preheated (42 °C) brucella broth and placed in an incubator at 42 °C. After this, 100 μL of the sample was taken every 10–120 min, and the titer was determined by plaque assay.

### 2.8 Host range, thermal, and pH stability

The host range was determined by spot assay (Akmal et al., 2023). A total of 56 *Campylobacter* isolates were checked (Supplementary File 1, Table S4). The stability of *Campylobacter* phage CC_R7 was assessed at different temperatures and pH levels. Briefly, phage suspension (10^7^ PFU/mL) was incubated at various temperatures, including 4 °C, 30 °C, 40 °C, 50 °C, 60 °C, 70 °C, and 80 °C. After incubation, the phage titer was determined at 0 min, 30 min, and 60 min. Phage stability against different pH levels (2–12) was determined by preparing solutions at the respective pH with Tris–chloride. Phage lysate (10^7^ PFU/mL) was added to different pH solutions and incubated at 37 °C. Samples were taken after 1 h, and the titer was determined by plaque assay.

### 2.9 Bacterial cell lytic assay (liquid medium)

To determine the bacterial cell lytic activity of phage in a liquid medium, the host *C. coli* (99-2) was grown in brucella broth at 42 °C for 24 h to attain 1 × 10^8^ CFU/mL. Then, 100 μL of bacteria and 100 μL of phage were added to a 100-well Honeycomb sterile microplate for bioscreen (Oy Growth Curves Ab Ltd) to attain MOI 0.1, 0.001, and 0.0001. In positive control, 200 μL of bacteria was added, and a blank well was used as a negative control. The plate was placed in a fully automatic growth curve analyzer (Bioscreen C, Oy Growth Curves Ab Ltd) at 42 °C, 220 rpm, and the OD 600 value was measured at 0 min and after every 30 min up to 24 h.

### 2.10 Effect of *Campylobacter* phage CC_R7 on biofilm eradication

The biofilm assay was performed by mimicking slaughterhouse conditions as described previously with slight modifications (Araújo et al., 2022). Given that the slaughter process comprises varying ambient temperature conditions, the biofilm formation was evaluated at 10 °C and 42 °C under microaerobic conditions. Chicken juice was prepared by taking 1 kg of chicken meat and 500 mL sterile saline solution and placing them in a sterile bag. The carcass fluid was collected and centrifuged at 12000 *g* for 15 min, and the supernatant was filtered through a 0.22 μm filter membrane. The host *C. coli* (99–2) was grown in brucella broth supplemented with 10 % chicken juice. 100 μL of phage suspension and 100 μL of host were mixed in a 96-well plate at MOI 100 and 10. In the control group, SM buffer was added, and plates were then incubated at 10 °C and 42 °C under microaerobic conditions. The crystal violet staining method was used to measure the formation of biofilm. Briefly, culture media were removed, and wells were washed 3 times with sterile Phosphate-buffered saline (PBS). After washing, methanol was added for 15 min, followed by air-drying. Stained with crystal violet (0.1 %) and 33 % glacial acetic acid was used for elution, with absorbance measured at OD value 595 nm (O'Toole, 2011).

### 2.11 *Campylobacter* phage CC_R7 application on chicken meat

Raw breast chicken meat was purchased from the supermarket in Wuhan, China. Chicken meat was sprayed with 70 % ethanol and washed with sterilized water for decontamination. The meat was cut into (2 cm × 2 cm) and put in UV light for 1 h on each side. Absence of *Campylobacter* was confirmed by homogenizing 1g of chicken in 10 mL PBS, and spreading 100 μL of this homogenate on a Columbia blood agar plate and observing the growth of *Campylobacter* colonies. After that, the meat surface was artificially contaminated with 100 μL of the host *Campylobacter* suspension (10^8^ CFU/mL) and incubated at room temperature for 1h to allow the bacterial cells to attach to the meat surface. Next, 100 μL purified phage suspension (2 × 10^9^ PFU/mL) was added to the surface of contaminated meat pieces and incubated at 4 °C. SM buffer was added to the control instead of the phage. After 0, 3, 6, 12, and 24 h, the sample was withdrawn and homogenized with PBS, and the homogenate was 10-fold serially diluted with PBS for viable count.

### 2.12 Statistical analysis

Statistical analysis was performed by using Statistical Package for the Social Sciences (SPSS) software version 22 (IBM, US). The infection dynamics tests were analyzed using GraphPad Prism 8.0.1 software. A significance level of p < 0.05 was employed for all statistical analyses. Each experiment was repeated three times, and data represent Mean ± standard error (Mean ± SEM).

## 3 Results

### 3.1 Bacterial isolation and antimicrobial susceptibility

153 *Campylobacter* isolates were included in this study. Of these, 120 (78.4 %) were *C. coli* and 33 (21.5 %) were *C. jejuni* (Table 1).

**Table 1:** The prevalence of *Campylobacter*.

Category	Isolates Count	Prevalence (%)
Total Samples	1120	
Total *Campylobacter*	153	13.3 %
*C. coli*	120	10.7 %
*C. jejuni*	33	2.9 %

*C. coli*: *Campylobacter coli, C. jejuni*: *Campylobacter jejuni*.

The antibiotic susceptibility test revealed that among the *C. coli* isolates, tetracycline has the highest rate of resistance (91.6 %), followed by nalidixic acid (90.8 %), ciprofloxacin (84.16 %), azithromycin (75.8 %), gentamycin (75 %), clarithromycin (65.8 %), erythromycin (38.3 %), telithromycin (29.1 %) and florfenicol (16.6 %). In contrast, ciprofloxacin (78.7 %) exhibited the highest resistance rate among the *C. jejuni* isolates. Nalidixic acid and tetracycline both had 75.75 % resistance compared to gentamycin (48.48 %), clarithromycin (27.27 %), azithromycin (10 %), erythromycin (6.1 %), and florfenicol (6.06 %). All *C. jejuni* isolates were sensitive to telithromycin. About 95 % (114/120) of *C. coli* and 85 % (28/33) of *C. jejuni* were multidrug-resistant (Table 2).

**Table 2:** Antimicrobial susceptibility of *C. coli* and *C. jejuni*.

Antibiotics	*C. coli* (n = 120) Total (% resistance)	*C. jejuni* (n = 33) Total (% resistance)
Ciprofloxacin	101 (84.16 %)	26 (78.7 %)
Nalidixic Acid	109 (90.8 %)	25 (75.75 %)
Gentamycin	90 (75 %)	16 (48.48 %)
Tetracycline	110 (91.6 %)	25 (75.75 %)
Clarithromycin	79 (65.8 %)	9 (27.27 %)
Erythromycin	46 (38.3 %)	2 (6.1 %)
Azithromycin	91 (75.8 %)	3 (10 %)
Telithromycin	35 (29.1 %)	0 (0 %)
Florfenicol	20 (16.6 %)	2 (6.06 %)
Multidrug-resistant	114 (95 %)	28 (85 %)

*C. coli*: *Campylobacter coli, C. jejuni*: *Campylobacter jejuni*.

### 3.2 Phage isolation and morphology

**Fig. 1:**
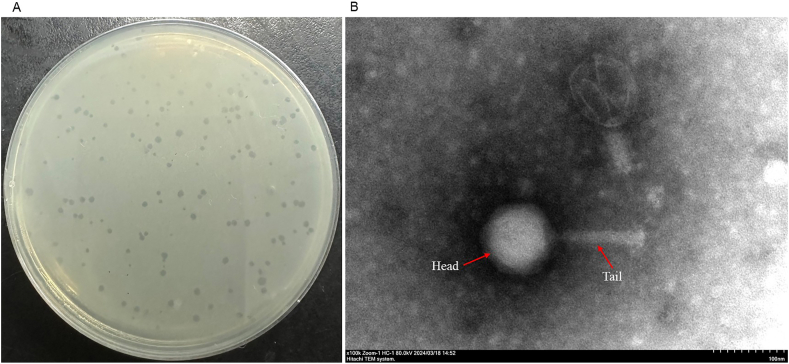
**Morphology of CC_R7**. (A) Plaque morphology of *Campylobacter* phage CC_R7 on the double agar plate. (B) Transmission electron microscope image of *Campylobacter* phage CC_R7. The phage belongs to the *Myoviridae* family with an icosahedral head and a long contractile tail. The diameter of the head was about 97.4 nm (±5.66 nm), and the tail length was approximately 126.8 nm (±3.2 nm).

A fecal sample collected from Hubei province confirmed the presence of lytic bacteriophage in a plaque assay. This *Campylobacter* phage, named CC_R7, showed a clear plaque morphology on a double agar plate measuring 1–2 mm (Fig. 1A). The transmission electron microscopic examination of *Campylobacter* phage CC_R7 (Fig. 1. B) revealed that it belongs to the *Myoviridae* family with an isometric head (diameter: 97.4 nm ± 5.66 nm) and a long contractile tail (length: 126.8 nm ± 3.2 nm).

### 3.3 Genomic analysis of *Campylobacter* phage CC_R7

The genomic analysis of *Campylobacter* phage CC_R7 confirmed a double-stranded DNA linear genome of 180,566 bp with a GC content of 27.8 %. The BLASTn results of the *Campylobacter* phage CC_R7 (PQ092953) nucleotide sequence showed that it has the highest similarity with the *Campylobacter* group II phages CP220 (Total score: 2.153e+05, Query cover: 83 %, Identity: 93.84 %). DNA Master (v5.23.6) predicted 200 ORFs and 2 tRNAs in the genome, with a coding capacity of 87.06 %. Among the predicted ORFs, 164 were on the positive strand, and 36 were on the negative strand. Of 200 ORFs, 87 (43.5 %) were assigned putative functions, whereas 113 ORFs (56.5 %) were not subjected to functional annotation. The genome contains three possible start codons. 175 ORFs use ATG, 16 ORFs use TTG, and 9 ORFs use GTG as a starting codon. Furthermore, phage carries two neighbouring tRNA genes: tRNA-Ile (GAT), specific for isoleucine amino acid, and tRNA-Tyr (GTA), specialized for the amino acid tyrosine.

**Fig. 2:**
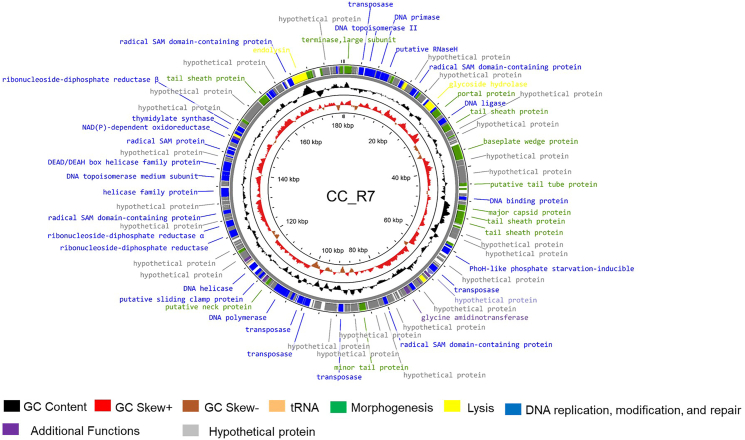
**Genomic map of Campylobacter phage CC_R7**. The products produced by ORFs are colour-coded according to their expected functions. The green colour is used to represent products that are involved in morphogenesis. Yellow is used to describe proteins that are involved in lysis. Blue is used to represent enzymatic proteins that are involved in DNA replication, modification, and repair. Purple is used to represent proteins with additional functions. Grey is used to represent hypothetical proteins. This map was created using Proksee.

A thorough examination of the recognized roles of various ORFs (Fig. 2) indicated the existence of several functional modules in the *Campylobacter* phage CC_R7, including those specific for morphogenesis, DNA modification, replication and repair, lysis, additional functions, and hypothetical proteins (Supplementary File 2, Detailed ORF annotation).

#### 3.3.1 Morphogenesis proteins

The morphogenesis module commenced with the large terminase protein and proteins involved in forming various head structures, including the major capsid protein, putative head completion protein, DNA end protector, and prohead protein protease. The tail protein included minor tail protein, baseplate protein, portal protein, and five genes encoding tail sheath protein, three genes encoding baseplate wedge subunit, two genes encoding tail tube protein, major tail protein, neck protein, and baseplate tube cap.

#### 3.3.2 Lysis proteins

The lysis proteins include glycoside hydrolase, polysaccharide deacetylase, UDP-glucose dehydrogenase, endolysin, a baseplate hub subunit, and a tail lysozyme, which is associated with the tail lysozyme and is responsible for the partial breakdown of the bacterial cell wall, allowing for phage DNA injection (Kanamaru et al., 2005).

#### 3.3.3 DNA replication, modification, and repair

The phage genome harbors numerous enzymatic proteins that play crucial roles in DNA replication, modification, and repair. These include DNA polymerase, DNA topoisomerase II, DNA primase, DNA helicase, SSB, DNA binding protein, sliding clamp loader large subunit, clamp loader small unit, RNaseH, DEAD/DEAH box helicase family protein, and PD-(D/E)XK nuclease family protein. This indicates that the phage relies on its machinery for replication. In addition to this, like other Group II phages, CC_R7 contains nine transposase genes, suggesting a high potential for causing deletions or genomic rearrangements. Moreover, as other Group II phages have radical SAM proteins, CC_R7 also has five radical SAM proteins. The Radical SAM superfamily comprises a wide range of enzymes with a common core structural fold and utilizes S-adenosylmethionine to produce organic radicals. Radical SAM enzymes catalyze reactions, including oxidation, reduction, methylation, methylthiolation, sulfurylation, and complicated rearrangement reactions (Farrar and Jarrett, 2007). Additionally, the genome has a 7-carboxy-7-deazaguanine synthase enzyme that shields the phage DNA from being targeted by the host's restriction enzymes (Hutinet et al., 2019). Phage CC_R7 has a multitude of genes that are implicated in nucleotide metabolism. The former includes aerobic and anaerobic ribonucleotide-diphosphate reductase genes, ribonucleoside-diphosphate reductase 1 subunit 1 ALPHA, ribonucleoside-diphosphate reductase subunit beta, thymidylate kinase, thymidine kinase, GTP cyclohydrolase I, and PhoH-like starvation inducible. As *Campylobacter* is microaerophilic, it may be inferred that phage CC_R7 possesses a repertoire of enzymes for synthesizing nucleotides in aerobic and anaerobic conditions.

The hypothetical protein module and additional functions modules are present in the remaining genome. No genes linked with lysogenicity, virulence, drug resistance, integrase, and pathogenicity were discovered, showing that phage CC_R7 was genetically safe. (Supplementary File 1. Table S5 summarizes the comparative properties of phage CC_R7 with those of other *Campylobacter* group II phages).

**Fig. 3:**
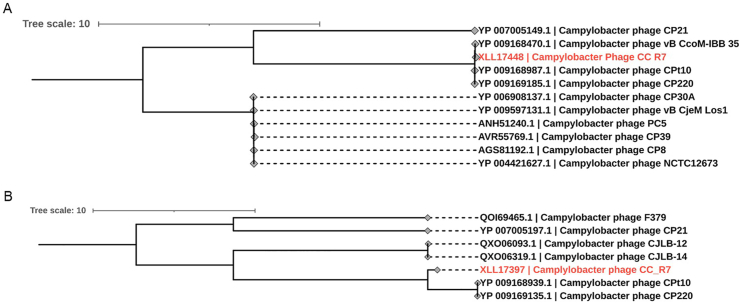
**Phylogenetic analysis of CC_R7.** (A) Neighbor-joining tree was constructed using the amino acid sequences of the major capsid protein (protein ID: XLL17448) and the (B) terminase large subunit (protein ID: XLL17397) of *Campylobacter* phage CC_R7 and similar protein sequences from other phages. The phylogenetic tree is accurately depicted, with branch lengths corresponding to the evolutionary distances utilized for its estimation. A bootstrap analysis was conducted using 1000 replicates. The terminal ends of branches are designated as "protein accession number followed by phage name." The evolutionary studies were performed using IQ-TREE v2.3.5.

The phylogenetic analysis (Fig. 3) of CC_R7 based on major capsid protein (Fig. 3A), and terminase protein (Fig. 3B) placed it closer to *Campylobacter* group II Phages (CP21, CP220, CPt10, and CcoM-IBB 35), which belong to the genus *Firehammervirus*.

**Fig. 4:**
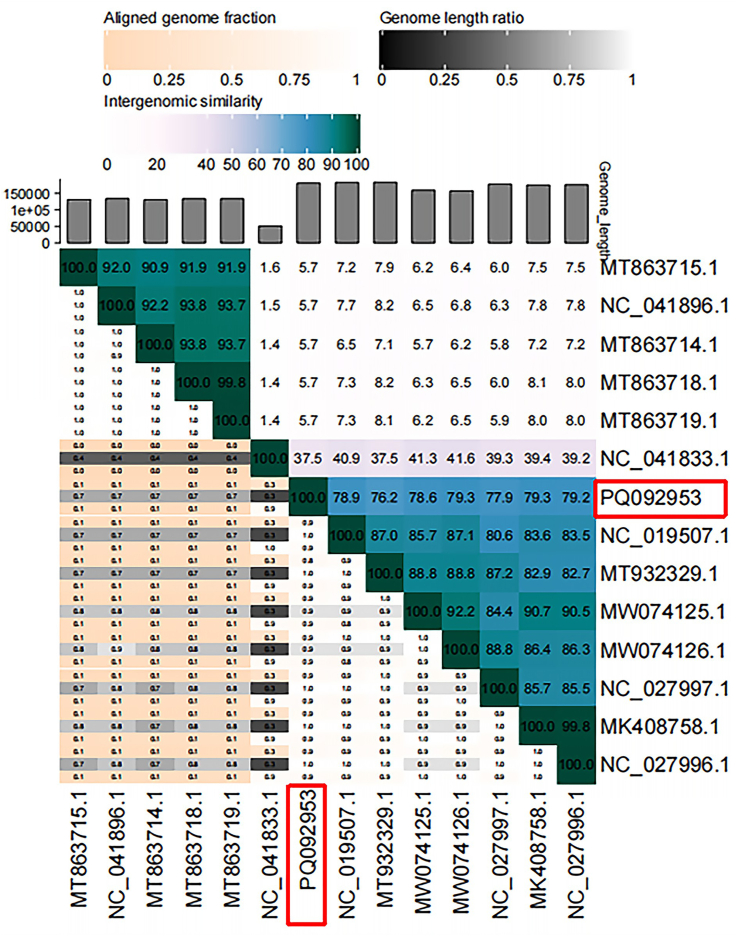
**VIRDIC analysis of CC_R7.** A heat map compares the entire genome of *Campylobacter* phage CC_R7 with 13 additional members belonging to the *Caudoviricetes* group. The heat map (similarity-based) was created using the VIRIDIC service, with a species differentiation threshold set at 95 %.

Furthermore, nucleotide-based intergenomic similarities as calculated with VIRDIC showed its clustering with other phages from the genus *Firehammervirus*, with the highest similarity (79.3 %) with the phage *Campylobacter* phage CP220, and *Campylobacter* phage CJLB-14 (Fig. 4).

**Fig. 5:**
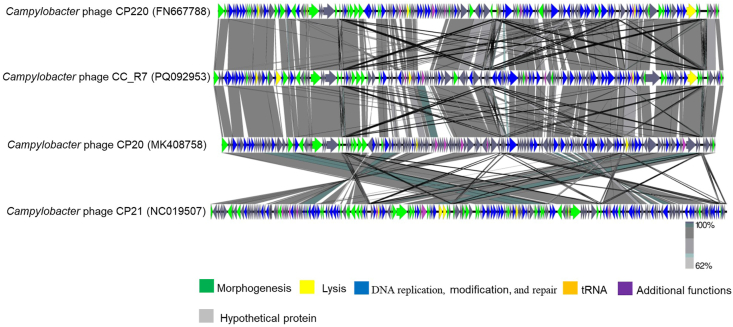
**Pairwise nucleotide sequence comparison of Campylobacter phage CP220 (FN667788), Campylobacter phage CC_R7 (PQ092953), Campylobacter phage CP20 (MK408758), and Campylobacter phage CP21 (NC019507) using tBLASTn.** The genomes are represented proportionally, with the scale bar indicating a length of 5000 bp. Arrows are used to denote the direction of transcription for each ORF. The products produced by ORFs are categorized and color-coded according to their projected functions, following the provided legend. The grey boxes indicate areas of genomic similarity, with the color gradient reflecting their level of identity.

The genomic comparison of *Campylobacter* phage CP220 (FN667788), *Campylobacter* phage CC_R7 (PQ092953), *Campylobacter* phage CP20 (MK408758), and *Campylobacter* phage CP21 (NC019507) by Easyfig (Fig. 5) revealed that these bacteriophages have greater symmetry and gene organization. The average nucleotide identity between CC_R7 and CP220 was calculated to be 91.71 %. Similarly, the average nucleotide identity between CC_R7 and CP20 was determined to be 91.34 %, and between CC_R7 and CP21, it was found to be 90.95 %.

### 3.4 Phage adsorption assay and one-step growth curve

**Fig. 6:**
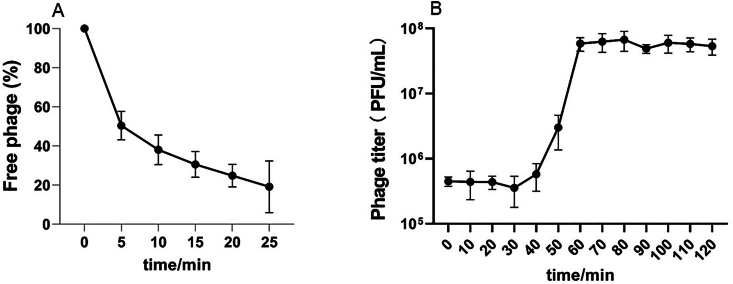
**Adsorption assay and one-step growth curve. (**A) Shows the rate at which *Campylobacter* phage CC_R7 adsorbs to its host. Phages were added to the bacterial suspension at an MOI of 0.1. The percentage of non-adsorbed/free phages was computed at the specified time points. The reported data are the average of three independent experiments, with error bars indicating the standard error of the mean (SEM). (B) Single-step growth curve study. Plaque-forming units (PFU) were measured at different intervals after infection with *C. coli* (99-2). The latent period is 40 ± 5 min, and the burst size is 119 ± 10 phages per infected cell. The bars represent SEM.

The *Campylobacter* phage CC_R7 was efficient in binding to its host *Campylobacter* strain. The decrease in free phage percentage in the supernatant after 5 and 10 min was about 50 % and 35 %, respectively, and continued to decrease until 25 min of incubation. The maximum adsorption was during the first 5 min, i.e., 50 % (Fig. 6A). The one-step growth curve experiment was performed to define the infection cycle of CC_R7, which showed that CC_R7 had a latent period of 40 ± 5 min and a burst size of roughly 119 ± 10 phages per infected cell (Fig. 6B). These findings strengthen the possibility that CC_R7 would be a helpful candidate for practical applications.

### 3.5 Host range, thermal, and pH stability

**Fig. 7:**
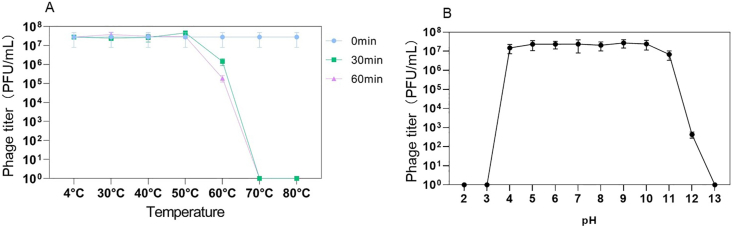
**Thermal and pH stability.** (A) Effect of temperature on the stability of *Campylobacter* phage CC_R7. The experiments were conducted three times, and the phage concentrations were reported as the average ± standard deviation. (B) Effect of pH on the stability of *Campylobacter* phage CC_R7.

The host range analysis showed that phage CC_R7 lysed 60 % (21/34) *C. coli* and 27.3 % (6/22) *C. jejuni*. The strongest lytic spectrum was observed in *C. coli* isolates isolated from Hubei. The host range of phage CC_R7, with varying degrees of lytic activity across different geographical regions is described in Supplementary File 1, Table S4. The *Campylobacter* phage CC_R7 is relatively stable at 50 °C. There was no significant change in titer from temperature 4 °C–50 °C. At 60 °C after 30 and 60 min, the titer decreases by 1 log and 2 log, respectively. At 70 °C, the phage was not detectable after 30 or 60 min (Fig. 7A). The *Campylobacter* phage CC_R7 is stable in a wide pH range from 4 to 11. In a highly acidic environment at pH 3, all phage particles lost infectivity. Similarly, in the highly basic environment at pH 12, the phage titer decreased sharply, and at pH 13, all phages were dead (Fig. 7B).

### 3.6 Bacterial cell lytic assay

**Fig. 8:**
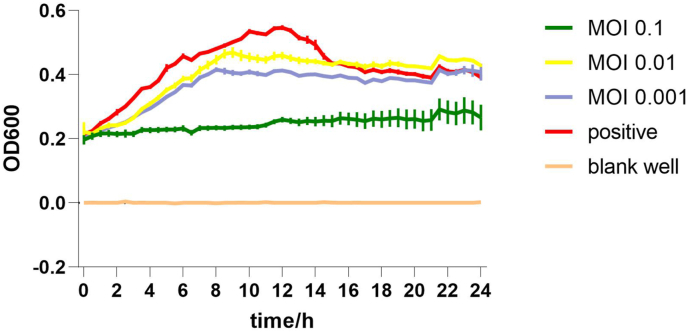
**Bacterial cell lytic assay.** The *Campylobacter* phage CC_R7 inhibits the growth of the host bacteria, *C. coli* (99-2). Phage was used at different MOIs. The positive control used SM buffer instead of phage. To detect *Campylobacter* growth, the OD 600 nm was measured. Results are presented as means ± SEM.

The lytic activity of *Campylobacter* phage CC_R7 in a liquid medium against its host bacteria at different MOIs is shown in Fig. 8. The maximum activity against bacteria is shown at MOI 0.1. The OD 600 value of positive control (without phage) and phage-treated (MOI 0.1) after 12 h reached 0.5 and 0.22, respectively. The host bacteria exhibited regrowth 21 h after infection, most likely attributed to the presence of spontaneous mutations (Fig. 8).

### 3.7 Effect of *Campylobacter* phage CC_R7 on biofilm eradication

**Fig. 9:**
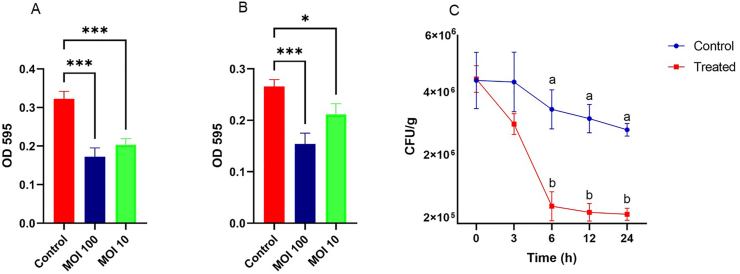
**Effect of Campylobacter phage CC_R7 on biofilm eradication at different temperatures. (A)** at 42 °C, **(B)** at 10 °C, **(C)** Effect of *Campylobacter* phage CC_R7 on the viability of host bacteria *C. coli* (99-2) on chicken meat. Values are expressed as mean ± SEM. Compared to the control group, ∗p < 0.05, and ∗∗∗p < 0.001. Different superscripts show statistically significant differences at p < 0.05.

The biofilm formation under different treatments was evaluated, as shown in Fig. 9. The control group (*Campylobacter* only) exhibited the highest biofilm formation, indicating robust biofilm development under microaerobic conditions at 42 °C (Fig. 9A). In contrast, the addition of phage CC_R7 at a **MOI of 100** resulted in a significant reduction in biofilm formation, demonstrating that phage at this concentration was effective in eradicating the biofilm. Similarly, the phage treatment at **MOI 10** also led to a reduction in biofilm formation, but this effect was less pronounced than at MOI 100. Similarly, at 10 °C (Fig. 9B), the control group exhibited higher biofilm formation as compared to the phage treatment groups.

### 3.8 *Campylobacter* phage CC_R7 application on chicken meat

The efficacy of the *Campylobacter* phage CC_R7 in eradicating or substantially reducing bacterial contamination on refrigerated (4 °C) retail chicken meat was assessed. The quantification of *Campylobacter* in the treated meat samples compared to the control samples was performed. A statistically significant (P < 0.05) difference was seen between the control and treated samples at 6, 12, and 24 h. The bacterial population in phage-treated chicken meat samples decreased by 1.2 logs CFU/g after 24 h (Fig. 9C).

## 4 Discussion

*Campylobacter* is one of the major foodborne pathogens on a global scale (Man, 2011). Poultry meat is widely recognized as a significant source of *Campylobacter* transmission (Shami et al., 2024). The irrational use of antibiotics in the poultry industry leads to the emergence of antibiotic resistance in *Campylobacter* (Barbol and Alsayeqh, 2024). It has increasingly become a significant public health concern in China and worldwide (Bai et al., 2021). Therefore, it is necessary to adopt enhanced management and preventative methods to regulate infections caused by MDR *Campylobacter* spp. effectively (Taha-Abdelaziz et al., 2023). To improve food safety and public health, *Campylobacter* phages can be used as a substitute for antibiotics to control foodborne *Campylobacter* pathogens (Chinivasagam et al., 2020; Zhang et al., 2024). Therefore, this study aimed to assess the antibiotic susceptibility of *C. coli* and *C. jejuni* strains from China. Additionally, the study aimed to isolate a novel lytic *Campylobacter* phage CC_R7 with a broad host range and to analyze its complete genomic sequence, characterization, and application in chicken meat processing. In this study, 153 *Campylobacter* strains were isolated from chicken feces. The prevalence of *C. coli* in chicken (78.4 %) was higher than that of *C. jejuni* (21.5 %). The antibiotic resistance rate in *C. coli* was higher than that observed in *C. jejuni* isolates from poultry. Moreover, these findings highlight a notably higher prevalence of MDR in both *C. coli* (95 %) and *C. jejuni* (85 %) (Tang et al., 2020; Gao et al., 2023).

In light of the growing problem of antibiotic resistance, phage therapy can be a potent tool against multidrug-resistant (MDR) food-borne infections like *Campylobacter* (Olson et al., 2022). Therefore, phage CC_R7 was isolated, which has a broad host range, lyses 60 % of *C. coli* and 22 % of *C. jejuni* of strains tested, surpassing the effectiveness of group II *Campylobacter* phage CP21 (Jäckel et al., 2015). Transmission electron microscopy analysis showed that CC_R7 belongs to the *Myoviridae* family with an isometric head and long contractile tail. The diameter of the head was 97.4 nm ± 5.66, and the length of the tail was 126.8 nm ± 3.2 nm. Therefore, it is consistent with *Campylobacter* phage group II (tail size 110–142 nm), which differs from group III phages (tail size 98–100 nm) in that it has a longer tail (Javed et al., 2014). The genome of *Campylobacter* phage CC_R7 (PQ092953) comprises 180,566 bp, placing it into the *Campylobacte*r phage group II. According to the International Committee on Taxonomy of Viruses, genus-level classification requires at least 70 % nucleotide identity across the whole genome, while species-level classification requires 95 % genome sequence identity (Adriaenssens and Brister, 2017). Phage CC_R7 could be included in the genus *Firehammervirus* because it shares the highest degree of similarity and genome organization with the *Campylobacter* phage CP220 as shown in Fig. 4, Fig. 5. The CC_R7 genome shared <95 % identity with *Campylobacter* phage CP220 (93.84 %) at the nucleotide level, as determined by BLASTn; therefore, CC_R7 may represent a new species in the genus *Firehammervirus*. The phage CC_R7 has a GC content of 27.8 %, which is lower than that of the host bacterium *Campylobacter* spp. (31 %), and approximately the same as that of other group II sequenced phages (CP220, CPt10, and CP21) (Timms et al., 2010; Jäckel et al., 2015). The phage CC_R7 primarily uses ATG as the primary initiation codon (87.5 %), consistent with the overall genetic makeup of bacterial and phage genomes reported in the NCBI (National Center for Biotechnology Information) (Belin, 1979; Villegas and Kropinski, 2008). Furthermore, CC_R7 contains tRNA genes for isoleucine (tRNA-Ile, GAT) and tyrosine (tRNA-Tyr, GTA), which sets it apart from previously sequenced group II *Campylobacter* phages (CP220, CPt10, and IBB_35) which have tRNAs for arginine (Arg) and tyrosine (Tyr) and phage (CP21) which has tRNAs for arginine (Arg) and proline (Pro). The presence of tRNA-Ile (GAT) in CC_R7 indicates that it has specifically evolved to improve translation in host conditions where isoleucine codons are common. This adaptation likely enhances the synthesis of viral proteins, replication efficiency, and infectivity for particular bacterial hosts (Jäckel et al., 2015). The sequence data obtained from CC_R7 provides information about the presence of morphogenesis proteins and a range of genes related to DNA replication, modification, and repair, lysis, and additional functions. At the protein level, most predicted CC_R7 products have over 90 % similarity to CP220 proteins. Furthermore, certain proteins have more than 20 % similarity with T4 bacteriophage (*Escherichia* phage T4, accession no. AF158101) proteins (supplementary File 1, Table S6) (Miller et al., 2003). Furthermore, CC_R7 does not contain any genes related to pathogenicity or virulence, making it genetically safe to use.

After the genomic analysis, phage CC_R7 underwent further categorization based on adsorption assay, burst size, phage stability, and lytic activity. The adsorption assay of CC_R7 showed good penetration power to host bacteria. The maximum (50 %) adsorption was during the first 5 min, showing its ability to effectively penetrate the host bacteria (Al-Mohammadi et al., 2022). Bacteriophage multiplication is demonstrated by its one-step growth curve, and optimizing phage fitness is linked to an ideal latent time, balancing it to enhance burst size. CC_R7 has a latent period of 40 min, which is shorter than that of phages phiCcoIBB35 (52.5 min), phiCcoIBB12 (82.5 min), and phiCcoIBB37 (67.5 min). The burst size of CC_R7 was 119 phages/infected bacteria, which is higher than that of phages phiCcoIBB35 (24 virions), phiCcoIBB12 (22 virions), and phiCcoIBB37 (9 virions) (Carvalho et al., 2010). In the food sector, it is preferable for bacteriophages utilized for biocontrol purposes to have large burst sizes and short latent periods (Kim et al., 2020). These findings provide evidence that bacteriophage CC_R7 has the potential to be a suitable candidate for practical applications.

Bacteriophages are specific to the receptors on the surface of host cells (Taslem Mourosi et al., 2022). Phage CC_R7 showed varying lytic activity across different regions of China, with the highest lytic activity observed in *C. coli* from Hubei. In Hubei, 9 out of 13 *C. coli* isolates were lysed (7 strongly, 2 weakly). In Hunan, 8 out of 13 *C. coli* isolates were lysed (5 strongly, 3 weakly), and in Jiangxi, 4 out of 8 were lysed (2 strongly, 2 weakly). For *C. jejuni*, 4 out of 10 isolates were lysed in Hubei (2 strongly, 2 weakly), while only weak lysis was observed in Hunan (1/7) and Jiangxi (1/5). These differences in lytic activity may be due to variations in the surface receptors of the bacteria, bacterial defense mechanisms, and regional genetic diversity (Holtappels et al., 2023).

Assessing the stability of bacteriophages at different temperatures and pH levels is crucial in determining their suitability for phage therapy applications (Ly-Chatain, 2014). This study showed that phage CC_R7 exhibited stability within the temperature range of 4 °C–50 °C. This feature allows the application of CC_R7 under different temperature conditions (Thung et al., 2020). Conversely, pH stability experiments demonstrated that the phage titer remained stable throughout a pH range of 4–10, showing its potential to work under different pH conditions (El-Shibiny et al., 2009).

Furthermore, the lytic potential of the phage CC_R7 against *Campylobacter* species was assessed at various MOIs. The viability of *Campylobacter* spp. infected with CC_R7 showed a substantial decrease compared to the untreated control. The most potent suppression of *Campylobacter* spp. occurred when the bacterial host was exposed to MOI 0.1. However, the observed re-growth of the host bacteria at 21 h after infection is probably caused by phage resistance due to spontaneous mutations; however, occurring at slower rate compared to previous research (Hwang et al., 2009).

To date, only one *Campylobacter* phage-based product (GRN 966) has received FDA approval for use in the meat industry, highlighting the need for further research to develop and approve additional phage-based interventions with a broader host range (FDA, 2020). Expanding the repertoire of well-characterized phage therapeutics could enhance their efficacy and applicability in controlling *Campylobacter*. In application, the phage CC_R7 showed a potential anti-biofilm activity against *Campylobacter* under slaughterhouse conditions, and the efficacy of phage CC_R7 was assessed in eradicating or substantially reducing bacterial contaminations on refrigerated (4 °C) retail chicken meat. In our result, the phage CC_R7 decreased the bacterial load by 1.2 logs on artificially contaminated chicken meat pieces at MOI (10). The CC_R7 treatment effectively stopped *Campylobacter* from growing at low temperatures, showing that the phage worked well against foodborne germs in cold conditions (Firlieyanti et al., 2016; Thung et al., 2020; Zhang et al., 2024).

## 5 Conclusion

The global rise of AMR *Campylobacter* in poultry meat is a major public health concern, with *C. coli* being more common and resistant to multiple drugs compared to *C. jejuni*. Researchers are exploring different strategies to address the issue of resistant *Campylobacter* contamination, such as phage therapy. The *Campylobacter* phage CC_R7 isolated in this study was successfully purified, and it has demonstrated significant lytic activity and stability. Genomic study indicates that phage CC_R7 has no genes linked to pathogenicity or resistance. The phage CC_R7 has shown potential in reducing *Campylobacter* levels in chicken meat, suggesting its usefulness as a biocontrol agent and offering an alternative to chemical bactericides. However, this study warrants further in-depth molecular typing or serotyping of the *Campylobacter* strains to better understand the host range of phage CC_R7, and in vivo analysis for the proper incorporation of *Campylobacter* phage CC_R7 as a potential biocontrol agent to control *Campylobacter* in the food chain.

## Credit authorship contribution statement

Muhammad Shahzad Rafiq: Conceptualization, Methodology, Investigation, Writing—original draft. Jie Chen, Muhammad Akmal, Dongxin Ma: Visualization, Writing—review & editing. Ali Asif, Junhao Wang: Data Curation. Yufeng Gu, Shuaifeng Gu: Writing—review & editing. Pan Tao: Methodology. Haihong Hao: Conceptualization, Supervision, Writing—review & editing.

## Data

The annotated complete genome sequence of *Campylobacter* phage CC_R7 reported herein is available at GenBank under accession PQ092953.

## Funding sources

This work was supported by the National Natural Science Foundation (NSFC) of China grant U23A20241/32172914, the Fundamental Research Funds for the Central Universities grant 2662025PY032, and the earmarked fund for CARS-41.

## Declaration of competing interest

The authors declare that they have no known competing financial interests or personal relationships that could have appeared to influence the work reported in this paper.

## Data Availability

Data will be made available on request.
