# Supplementary material for: Characterization and application of a novel Campylobacter phage CC_R7 as a biocontrol agent in chicken meat
Source: Curr Res Food Sci. 2025 Aug 20;11:101182. doi: 10.1016/j.crfs.2025.101182 (PMC12398825; doi:10.1016/j.crfs.2025.101182)
Supplement: Multimedia component 1 [file mmc1.docx]

**Electronic Supplementary Material**

**Characterization and Application of a Novel *Campylobacter* Phage CC_R7 as a Biocontrol agent in Chicken meat.**

**Muhammad Shahzad Rafiq^1, 2^, Jie Chen^1, 2^, Muhammad Akmal^5^, Dongxin Ma^1, 2^ , Ali Asif^1,2^, Junhao Wang^1,2^ , Shuaifeng Gu^1,2^, Yufeng Gu^1, 2^, Pan Tao^1,^ , Haihong Hao^1, 2, 3, 4*^**

^1^National Key Laboratory of Agricultural Microbiology, Huazhong Agricultural University, Wuhan, China;

^2^National Reference Laboratory of Veterinary Drug Residues, Huazhong Agricultural University, Wuhan 430070, China;

^3^Huazhong Agricultural University, Shenzhen Institute of Nutrition and Health, Shenzhen 518000, China;

^4^Shihezi University, Shihezi City, Xinjiang 832000, China.

^5^Department of Fisheries and Aquaculture, University of Veterinary and Animal Sciences, Lahore 54000, Pakistan

**^*^Correspondence:** Haihong Hao

Tel.; 008615871812208

Email: haohaihong@aliyun.com

**Table S1. *Campylobacter* strains used to isolate phage**

| **Strain No** | **Strain Type** | **Strain No** | **Strain Type** |
| --- | --- | --- | --- |
| 146-1 | *C. coli* | TMXF6 | *C. jejuni* |
| 132-2 | *C. coli* | PDC6 | *C. jejuni* |
| 112-1 | *C. coli* | YCCW2 | *C. jejuni* |
| XS1 | *C. coli* | TMXF4 | *C. jejuni* |
| 1-6 | *C. coli* | TMXF1 | *C. jejuni* |
| 156-1 | *C. coli* | FXC24 | *C. jejuni* |
| 123-1 | *C. coli* | J30 | *C. jejuni* |
| 7-1 | *C. coli* | FXC10 | *C. jejuni* |
| 163-2 | *C. coli* |  |  |

*C. coli: Campylobacter coli, C. jejuni: Campylobacter jejuni*

**Table S2. Primers used to find the gap/missing sequence between two contigs**

| **Target** | **Names** | **Primer Sequence 5’-3’** |
| --- | --- | --- |
| Primer set 1 | 1F-primer | AAATGCGGACATTGTTGTG |
|  | 1R-primer | TTTTTTCGGGGTTTTAAACC |

The total volume of the PCR reaction was 50 µL. The mixture comprised 5 µL DreamTaq buffer, 5 µL DNTP solution, 1.25 µL DreamTaq polymerase enzyme, 24.75 µL RNase-free water, F-primer 5 µL, R-primer 5 µL, and DNA 4 µL/ 25ng. Reaction condition comprised initial denaturation at 94 °C for 2 min followed by 35 cycles, including denaturation at 94 °C for 15s, annealing at 50 °C for 15s, extension at 72 °C for 210s, followed by a final extension at 72 °C for 1 min.

**Table S3. Genome closure primers**

| **Target** | **Names** | **Primer Sequence 5’-3’** |
| --- | --- | --- |
| Primer set 4 | 4F-primer | CTGGACTTGATAGTGTATCTTTGATGG |
|  | 4R-primer | CTACGCACAGTTATACCGATGTTATC |

The total volume of the PCR reaction was 50 µL. The mixture comprised 5 µL DreamTaq buffer, 5 µL DNTP solution, 1.25 µL DreamTaq polymerase enzyme, 24.75 µL RNase-free water, F-primer 5 µL, R-primer 5 µL, and DNA 4 µL/ 25ng. Reaction condition comprised initial denaturation at 94 °C for 2 min followed by 35 cycles, including denaturation at 94 °C for 15s, annealing at 47 °C for 15s, extension at 72 °C for 210s followed by a final extension at 72 °C for 1 min.

**Table S4. Host range of *Campylobacter* phage CC_R7**

| **Strain No** | **Area** | **Strain** | **Lytic Activity** | **Strain No** | **Area** | **Strain** | **Lytic Activity** |
| --- | --- | --- | --- | --- | --- | --- | --- |
| 132-2 | Hubei | *C. coli* | ++ | CC-010 | Hunan | *C. coli* | - |
| CC-HBXG-1 | Hubei | *C. coli* | ++ | CC-011 | Hunan | *C. coli* | ++ |
| Y-SH-25 | Hubei | *C. coli* | ++ | LHN-11 | Hunan | *C. coli* | - |
| XS1 | Hubei | *C. coli* | ++ | XY-43 | Hunan | *C. coli* | ++ |
| CC-031 | Hubei | *C. coli* | + | XY-5 | Hunan | *C. coli* | + |
| T-GH-26 | Hubei | *C. coli* | - | CC-020 | Hunan | *C. coli* | + |
| Y-CW-26 | Hubei | *C. coli* | - | LH-39 | Jiangxi | *C. coli* | - |
| 123-1 | Hubei | *C. coli* | ++ | 112-1 | Jiangxi | *C. coli* | + |
| 146-1 | Hubei | *C. coli* | ++ | 1-6 | Jiangxi | *C. coli* | - |
| CC-HBXG-2 | Hubei | *C. coli* | - | 7-1 | Jiangxi | *C. coli* | - |
| T-GH-18 | Hubei | *C. coli* | - | 163-2 | Jiangxi | *C. coli* | - |
| CC-021 | Hubei | *C. coli* | ++ | YCTTY3 | Jiangxi | *C. coli* | + |
| Y-SH-56 | Hubei | *C. coli* | + | Sw-02 | Jiangxi | *C. coli* | ++ |
| LH-40 | Hunan | *C. coli* | - | XC-26 | Jiangxi | *C. coli* | ++ |
| XY-23 | Hunan | *C. coli* | + | Y-MD-6 | Hubei | *C. jejuni* | + |
| 156-1 | Hunan | *C. coli* | - | PDC5 | Hubei | *C. jejuni* | - |
| CC-HNZZ-5 | Hunan | *C. coli* | ++ | FC11168 | Hubei | *C. jejuni* | + |
| xc-48 | Hunan | *C. coli* | ++ | FXC1 | Hubei | *C. jejuni* | - |
| XY-11 | Hunan | *C. coli* | - | PDC15 | Hubei | *C. jejuni* | - |
| XC-33 | Hunan | *C. coli* | ++ | LUX-02 | Hubei | *C. jejuni* | - |
| FXC24 | Hubei | *C. jejuni* | - | TMXF4 | Hunan | *C. jejuni* | - |
| J30 | Hubei | *C. jejuni* | - | LH-21 | Hunan | *C. jejuni* | - |
| PDC6 | Hubei | *C. jejuni* | ++ | TMGH | Hunan | *C. jejuni* | + |
| FXC10 | Hubei | *C. jejuni* | ++ | YCMD1 | Jiangxi | *C. jejuni* | - |
| T-GH-18 | Hunan | *C. jejuni* | - | YCCW2 | Jiangxi | *C. jejuni* | - |
| TMXF1 | Hunan | *C. jejuni* | - | X-k-04 | Jiangxi | *C. jejuni* | - |
| TMXF6 | Hunan | *C. jejuni* | - | YCCW02 | Jiangxi | *C. jejuni* | + |
| TMXF5 | Hunan | *C. jejuni* | - | LH-03 | Jiangxi | *C. jejuni* | - |

*C. coli: Campylobacter coli, C. jejuni: Campylobacter jejuni*, ++: phage strong lysed the bacteria or clear lysis, +: phage weakly lysed the bacteria or turbid plaque, -: phage did not lysed the bacteria.

**Table S5. Comparative properties of phage CC_R7 with other *Campylobacter* group II phages**

| **Phage** | **CP220** | **CPt10** | **CP21** | **IBB_35** | **CC_R7** |
| --- | --- | --- | --- | --- | --- |
| Source | Chicken | Environment | Water organic farm | Poultry ceca | Chicken faeces |
| Isolation year | 2003 | 1989 | 2011 | N/A | 2023 |
| Country | United Kingdom | United Kingdom | Germany | Portugal | China |
| Sequencing | Shotgun seq. DNA libraries | 454 FLX pyroseq. and PCR/ Sanger | 454 FLX pyroseq., and PCR/ Sanger | 454 FLX pyroseq. | Shotgun seq DNA libraries and PCR sanger |
| Host species | *C. coli,*  *C. jejuni* | *C. coli,*  *C. jejuni* | *C. coli,*  *C. jejuni* | *C. coli,*  *C. jejuni* | *C. coli,*  *C. jejuni* |
| Family | *Myoviridae* | *Myoviridae* | *Myoviridae* | *Myoviridae* | *Myoviridae* |
| Head diameter(nm) | 96 | N/A | 93 | 100 | 97.4 |
| Tail size (nm) | 110 x 16 | N/A | 135 x 24 | 140 x 17 | 126.8 |
| Genomic size (bp) | 177,493 | 175,720 | 182761 | 172,065 | 180,566 |
| GC Content (%) | 27.4 | 27.3 | 27.2 | 27.4 | 27.8 |
| Coding Capacity (%) | 88.4 | 89.7 | 88.2 | 90 | 87.06 |
| Total ORFs | 194 | 201 | 220 | 210 | 200 |
| ORFs with functions | 74 (38%) | 70 (35%) | 77 (35%) | 84 (40%) | 87 (43.5%) |
| Forward strand | 173 | 180 | 91 | N/A | 164 |
| Reverse strand | 21 | 21 | 129 | N/A | 36 |
| tRNAs | 2 (Arg, Tyr) | 2 (Arg, Tyr) | 2 (Thr, Pro) | 2 (Arg, Tyr) | 2 (Ile, Tyr) |
| SAM-related proteins | 11 | 12 | 8 | 6 | 5 |
| Transposase | 4 | 2 | 5 | 2 | 9 |
| T4-Type proteins | 39 | 34 | 32 | 35 | 42 |
| Genome structure | Linear | Linear | Linear | N/A | Linear |
| Sequence complete | Complete | Complete | Complete | Incomplete | Complete |
| Accession no | FN667788 | FN667789 | NC019507 | HM246720-4 | PQ092953 |
| Reference | (Timms et al., 2010) | (Timms et al., 2010) | (Hammerl et al., 2012) | (Carvalho et al., 2012) | This study |

N/A: not available, *C. coli: Campylobacter coli, C. jejuni: Campylobacter jejuni,* SAM: S-adenosylmethionine, tRNA: transfer ribonucleic acid, PCR: polymerase chain reaction.

**Table S6. Comparison of different proteins of CC_R7, T4 bacteriophage and CP220**

| **CC_R7** | | **CC_R7/T4 identity %** | | **CC_R7/CP220 identity %** | |
| --- | --- | --- | --- | --- | --- |
| **Gene No** | **Gene function** | **T4 gene NO** | **Identity %** | **CP220 gene no** | **Identity %** |
| ORF1 | Terminase, large subunit | *gp17* | 32% | CPT_0001 | 94% |
| ORF6 | DNA topoisomerase II | *gp39* | 35% | CPT_0005 | 97% |
| ORF10 | DNA primase | *gp61* | 28% | CPT_0009 | 92% |
| ORF11 | Clamp loader large subunit | *gp44* | 30% | CPT_0010 | 98% |
| ORF12 | Putative RNaseH | *Rich* | 37% | CPT_0011 | 99% |
| ORF29 | Recombination endonuclease VII | *gp49* | 25% | CPT_0029 | 88% |
| ORF30 | Portal protein | *gp 20* | 29% | CPT_0030 | 98% |
| ORF32 | DNA ligase | *gp30* | 27% | CPT_0033 | 96% |
| ORF34 | Tail sheath protein | *gp18* | 24% | CPT_0034 | 97% |
| ORF36 | Baseplate wedge subunit | *gp25* | 39% | CPT_0037 | 98% |
| ORF42 | Baseplate wedge protein | *gp6* | 21% | CPT_0041 | 95% |
| ORF46 | putative tail tube protein | *gp19* | 28% | CPT_0045 | 98% |
| ORF47 | Putative head completion protein | *gp4* | 31% | CPT_0046 | 96% |
| ORF49 | DNA binding protein | *gp55* | 27% | CPT_0048 | 98% |
| ORF52 | Putative major capsid protein | *gp23* | 31% | CPT_0051 | 95% |
| ORF54 | Tail sheath protein | *gp18* | 30% | CPT_0053 | 96% |
| ORF59 | Major tail protein | *gp19* | 24% | CPT_0058 | 100% |
| ORF116 | DNA polymerase | *gp43* | 28% | CPT_0115 | 97% |
| ORF127 | DNA helicase | *gp41* | 27% | CPT_0125 | 99% |
| ORF154 | Helicase family protein | *UvsW* | 30% | CPT_0148 | 97% |
| ORF177 | Tail sheath protein | *gp15* | 26% | CPT_0174 | 96% |
| ORF180 | SSB protein | *gp32* | 31% | CPT_0177 | 94% |
| ORF199 | Prohead core protein protease | *gp21* | 34% | CPT_0193 | 96% |
| ORF 139 | Ribonucleoside-diphosphate reductase 1 subunit 1 ALPHA | *nrdA* | 31% | CPT_0134 | 95% |
| ORF172 | Ribonucleoside-diphosphate reductase subunit beta | *nrdB* | 31 | CPT_0168 | 99% |
| ORF72 | baseplate hub subunit and tail lysozyme | *gp5* | 25% | - | - |

DNA**: deoxyribonucleic acid,** SSB protein**: Single-Strand Binding protein.**
